# Comparison of the clinical and prognosis risk factors between endoscopic resection and radical gastrectomy for early-stage gastric cancer

**DOI:** 10.1186/s12957-023-03018-5

**Published:** 2023-05-12

**Authors:** Qianze Dao, Ke Chen, Liang Zhu, Xiaoshan Wang, Mengding Chen, Jian Wang, Zhengguang Wang

**Affiliations:** 1 Department of General Surgery, the First Affiliated Hospital of Anhui Medical University, Jixi Road, Hefei, Anhui Province China; 2 Drum Tower Hospital Affiliated to Medical School, Nanjing University, Nanjing, Jiangsu Province China; 3 Department of General Surgery, Anhui Provincial Hospital, Hefei, Anhui Province China

**Keywords:** Early gastric cancer, Endoscopic submucosal dissection, Endoscopic mucosal resection, Laparoscopic-assisted radical gastrectomy, Open radical gastrectomy

## Abstract

**Aim:**

This study aimed to explore the efficacy and safety of endoscopic submucosal dissection/endoscopic mucosal resection (ESD/EMR), laparoscopic-assisted radical gastrectomy (LARG), and open radical gastrectomy (ORG) in early-stage gastric cancer.

**Methods:**

A total of 417 patients with early-stage gastric cancer who were admitted in two hospitals from January 1, 2014 to July 31, 2017 were selected; the patients were divided into the ESD/EMR group (139 cases), LARG group (108 cases), and ORG group (170 cases) according to the operation methods used. The baseline data, economic cost of health, oncologic characteristics, postoperative complications, 5-year overall survival and disease-free survival, and risk factors of death were compared and analyzed.

**Results:**

No significant difference was observed in the baseline data among the three patient groups (*P* > 0.05). The total hospitalization days, operation time, postoperative fluid intake time, hospitalization expenses, and proportion of antibiotic use rate in the ESD/EMR group were lesser than those in other groups (*P* < 0.05). The LARG group has a longer operation time and higher hospitalization expenses compared with the ORG group (*P* < 0.05), but the total hospitalization days, postoperative fluid intake time, proportion of antibiotic use, and lung infection status were consistent. The ESD/EMR group had a lower incidence of incision site infection and postoperative abdominal distension compared with that of the surgery groups (*P* < 0.05). Five patients required radical surgery after undergoing ESD/EMR (The patients had residual tissue margin cancer), while none of the patients had switched to ORG during LARG. Surgery had advantages over ESD/EMR in terms of lymph node dissection (*P* < 0.05). No significant differences were observed in the postoperative complications such as upper gastrointestinal bleeding, perforation, incision hernia, reoperation and recurrence (*P* > 0.05). The 5-year postoperative survival rates of patients in the three groups were 94.2% (ESD/EMR), 93.5% (LARG), and 94.7% (ORG), respectively, with no significant differences (*P* > 0.05). The binary logistics multivariate analysis showed that the tumor size, invasion depth, vascular invasion, and differentiated degree were risk factors for death in patients with gastric cancer.

**Conclusions:**

No significant difference was observed between ESD/EMR and radical surgery. However, standardized criteria for excluding metastatic lymph nodes should be established to promote ESD/EMR.

Gastric cancer is a type of cancer occurring in the digestive system, the epithelial cells of the gastric mucosa become cancerous. According to relevant data, 1 million new cases of gastric cancer were reported worldwide, and 800,000 people died from gastric cancer each year [1, 2]. According to the depth of tumor tissue invasion, gastric cancer can be divided into advanced-stage gastric cancer and early-stage gastric cancer. In advanced-stage gastric cancer, the tumor tissue invasion exceeds the submucosal layer and enters the muscular layer. Advanced-stage gastric cancer had a poor prognosis, and the 5-year survival rate was 20%–25% [3]. Early-stage gastric cancer (EGC) refers to tumor invasion limited to the mucosa and submucosa, regardless of lesion size and lymph node metastasis [4], its prognosis was good, and the 5-year survival rate was higher than 90% [5].

At present, the common EGC treatment methods include Endoscopic submucosal dissection (ESD) or Endoscopic mucosal resection (EMR), laparoscopic-assisted radical gastrectomy (LARG), and open radical gastrectomy (ORG). To date, the EGC treatment methods remain controversial, and only a few comparative studies had reported the clinical prognosis of ESD and radical surgery in EGC. This study analyzed the clinical prognosis of EGC treated by ESD/EMR, LARG, and ORG, in order to provide reference for clinical selection of reasonable treatment.

## Methods

### Patients

**Fig. 1:**
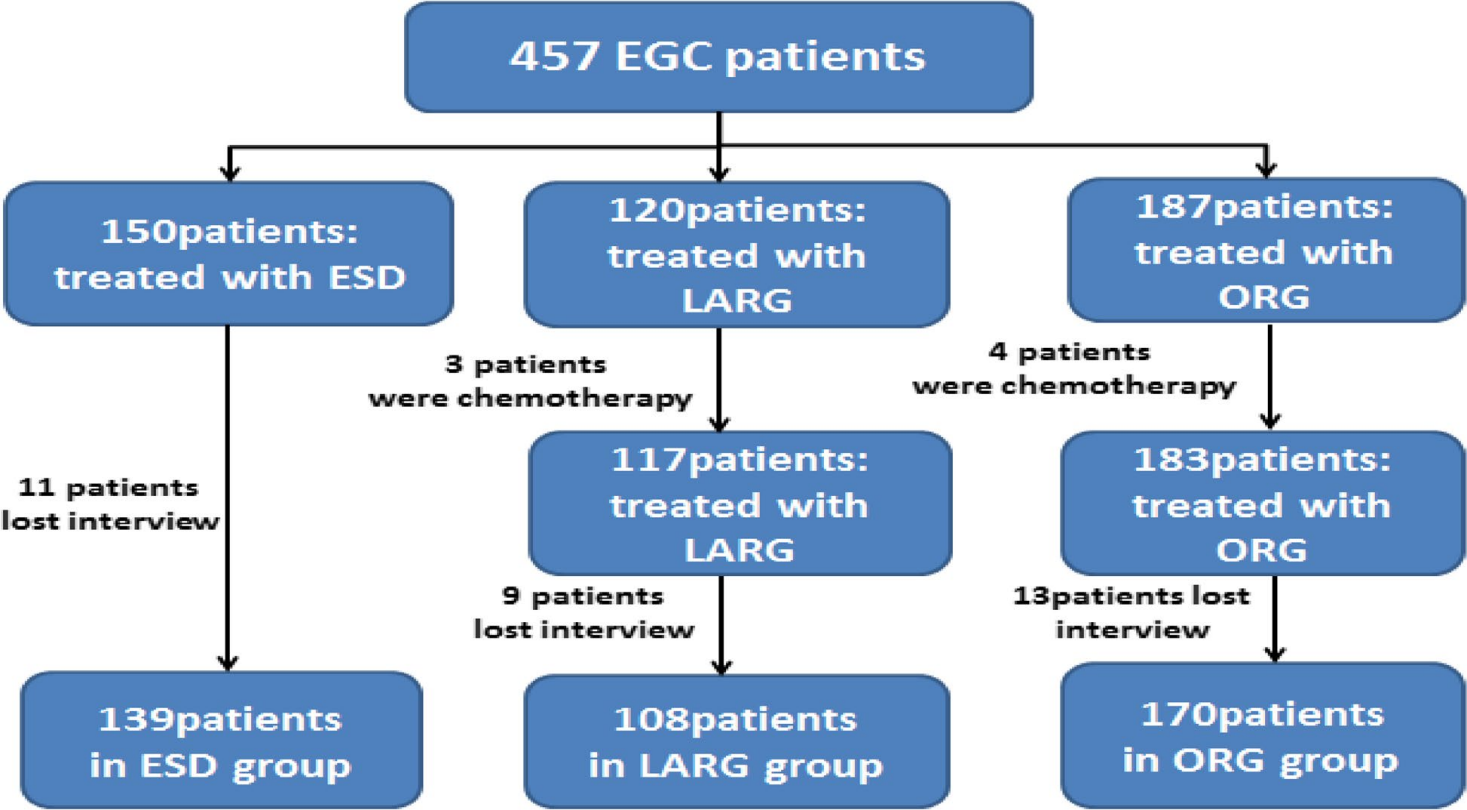
Flowchart of the process of screening gastric cancer patients

EGC patients admitted in the First Affiliated Hospital of Anhui Medical University and Anhui Provincial Hospital from January 1, 2014 to July 31, 2017 were included in the study. The postoperative pathological results of patients clearly showed invasion of cancer in the mucosa and submucosa. Patients who received chemotherapy and radiotherapy before surgery and who were lost to follow-up were excluded; the screening process is shown in Fig. 1. Follow-up time was defined as the period from the first postoperative day until death or end of follow-up; the follow-up deadline was set to July 31, 2022. The study was approved by the Ethics Committee of the First Affiliated Hospital of Anhui Medical University and Anhui Provincial Hospital.

### ESD/EMR

All patients included in the study completed endoscopic evaluation before surgery, the endoscopic evaluation was based on white light endoscopy combined with image enhanced endoscopy. Enhanced abdominal CT or PET-CT examination was performed to exclude regional lymph node metastasis. Moreover, endoscopic ultrasonography (EUS) examination had a very important guiding significance for evaluating the depth of invasion in early gastric cancer, and it can also be used to evaluate regional lymph node metastasis. According to our evaluation, the patients included in the study should meet the absolute and expanded indications for ESD/EMR therapy. The absolute indications include differentiated intramucosal carcinomay without ulceration and differentiated intramucosal carcinoma with a lesion size of ≤ 3 cm. Meanwhile, the expanded indications include 1. undifferentiated intramucosal carcinoma with a lesion size of ≤ 2 cm and no ulceration and 2. differentiated adenocarcinoma with a submucosal infiltration depth of no more than 500 um and a diameter of ≤ 3 cm. Exclusion criteria: 1. Early gastric cancer patients who do not meet ESD/EMR surgical indications. 2. Patients with poor general condition, complicated with serious cardiopulmonary insufficiency and malignant tumors of other organs.

#### ESD

Under general anesthesia, methylene blue dye was injected within 5 mm outside the tumor edge; after successful labeling, the mixture (250 ml glycerol fructose, 1 ml epinephrine, and 1 ml methylene blue) was submucosally injected to mark the edges and better define the mucous membrane after lifting the tumor. An IT knife was used to cut and uplift the tag along the edges, between the submucosa and muscularis, until complete resection of the neoplasm was achieved. Intraoperative bleeding was electrocoagulated, and the perforation was clipped with titanium.

#### EMR

Most of the patients had differentiated intramucosal carcinoma of < 2 cm in diameter. A mixture of methylene blue and glycerol fructose was injected into the submucosa around the lesion to raise the lesion. The high-frequency snare was installed in the transparent cap, and then the transparent cap was placed in front of the endoscope. The lesion mucosa was sucked into the transparent cap by negative pressure attraction and then cut with the snare. For large lesions that could not be resected at one time, the lesions were dissected and removed, and hemostasis was stopped by spraying hemostatic drugs and using electrocoagulation; titanium clips were applied when necessary.

### Gastrectomy

#### LARG

All patients included in the study completed endoscopic evaluation before surgery. Enhanced abdominal CT or PET-CT examination was performed to exclude regional lymph node metastasis. According to our evaluation, the patients included in the study should meet the absolute and expanded indications for ESD/EMR therapy. General anesthesia was administered by endotracheal intubation, the patient was positioned supine on the operating table, and the five-step method was used to perform the procedure; pneumoperitoneum was established, with the pressure was set at 10–15 mmHg. In order to perform a laparoscopic-assisted gastric lymph node dissection, a 6–8 cm auxiliary incision was made below the median xiphoid process of the upper abdomen. The tumor and stomach were excised, and digestive tract reconstruction was performed. A drainage tube was placed under the liver, and a drainage tube was placed in the abdominal cavity.

#### ORG

All patients included in the study completed endoscopic evaluation before surgery. Enhanced abdominal CT or PET-CT examination was performed to exclude regional lymph node metastasis. According to our evaluation, the patients included in the study should meet the absolute and expanded indications for ESD/EMR therapy. The patient was placed in supine position, and the procedure was performed under general anesthesia through endotracheal intubation. Then, a 15–20-cm left incision around the umbilicus of the subxiphoid midline was made to separate the tissue layer by layer. The abdominal cavity of the patient was opened to expose the surrounding gastric tissue. The patient had antral cancer and required radical distal gastrectomy. Radical gastrectomy was performed if the patients had cancer in the fundus, cardia, and body of the stomach; the specimens of the stomach were removed, and digestive tract reconstruction was carried out. A drainage tube was placed under the liver, and a drainage tube was placed in the abdominal cavity.

### Evaluation of short-term clinical efficacy

The patients baseline information was collected from the medical records and evaluated to determine whether their baseline data were comparable. The clinical and pathological results were further collected, and tumor location was determined based on the surgical findings. The tumor size and depth of invasion were determined based on the postoperative pathological results. The total hospitalization cost, operation time, total length of hospital stay, postoperative hospital stay, bleeding, and other relevant information were collected by referring to relevant case data.

Definition of related indicators: The total length of hospital stay was defined as the time from admission to discharge; bleeding was defined as the continuous flow of dark red fluid from the gastric tube. There was obvious oozing of blood in the visual field during the operation and was stopped by endoscopic techniques, operation, or drug treatment, blood transfusion was performed after surgery. Perforation was defined as the presence of free gas below the diaphragm on postoperative X-ray.

### Follow-up

The patients were followed up to assess whether long-term postoperative complications and recurrence occurred after surgery; the 5-year overall survival (OS) and disease-free survival (DFS) were also assessed. After discharge, outpatient follow-up was performed every 6 months for the first 2 years and annually thereafter. Outpatient or telephone follow-up was conducted 5 years later. Results of endoscopy, abdominal CT, blood routine, biochemical, and blood tumor marker tests were reviewed periodically during follow-up. OS was defined as the duration from the first postoperative day to death from any cause. DFS was defined as the time from the first day after surgery to recovery or death from any cause.

### Statistics

SPSS25.0 software was used for statistical processing; the measurement data were expressed as M (P25 and P75) and analyzed using multiple rank sum test. Counting data were expressed as rate (%) and analyzed using chi-square test. The Kaplan–Meier method was used for survival analysis. *P* value of < *0.0*5 was considered significant.

## Results

### Comparative analysis of baseline data

According to the above criteria, a total of 417 participants were selected including 310 men and 107 women; the participants were divided into three groups according to the treatment methods used: ESD/EMR group (139), LARG group (108), and ORG group (170). Patients were followed up from 2 to 102 months, with a median follow-up of 72 months. No significant differences were observed in gender, age, body weight, preoperative blood routine indexes, blood albumin levels, smoking and drinking history, cardiovascular and cerebrovascular history, diabetes history, family history of cancer, and blood tumor markers (alpha fetoprotein, carcinoembryonic antigen, cancer antigen 125, and cancer antigen 19–9) among the three groups of gastric cancer patients (*P* > 0.05). The general conditions of the three groups of gastric cancer patients were similar and comparable, as shown in Table 1.

**Table 1:** Comparative analysis of baseline data

Characteristic	ESD/EMR (*n* = 139)	LARG (*n* = 108)	ORG (*n* = 170)	*P*
Sex (%)				0.07
Male	113 (81.3)	76 (70.3)	121 (71.2)	
Female	26 (18.7)	32 (29.7)	49 (28.8)	
Year (%)				0.21
≥ 60	91 (65.5)	59 (54.6)	101 (59.0)	
< 60	48 (34.5)	49 (45.4)	69 (41.0)	
Smoking (%)	24 (17.3)	22 (20.4)	31 (18.2)	0.47
Drink (%)	14 (10.0)	13 (12.0)	18 (10.6)	0.88
Angiosis (%)	26 (18.7)	19 (17.6)	31 (18.2)	0.97
Diabetes (%)	5 (3.6)	3 (2.8)	7 (4.1)	0.94*
Family history (%)	4 (2.9)	3 (2.8)	5 (2.9)	0.99*
Weight (kg)	60 (55–68)	63 (58–68)	61 (55–68)	0.32
WBC (10^9^/ml)	5.4 (4.8–5.5)	5.4 (4.9–6.2)	61 (55–68)	0.32
NE (10^9^/ml)	2.9 (2.4–3.7)	3.31 (2.5–4.0)	3.1 (2.6–3.9)	0.13
HB (g/L)	133 (124–143)	135 (122–145)	133 (120–144)	0.64
RBC (10^12^/ml)	4.4 (4.1–4.7)	4.4 (4.1–4.7)	4.4 (4.1–4.7)	0.95
PLT (10^9^/ml)	189 (155–234)	197 (159–241)	182 (145–221)	0.10
ALB (g/L)	42 (40–45)	43 (40–45)	43 (40–46)	0.47
AFP (ng/ml)	2.2 (1.9–3.0)	2.2 (1.6–3.4)	2.4 (1.6–3.3)	0.76
CEA (ng/ml)	2.1 (1.3–3.0)	2.3 (1.3–3.3)	1.9 (1.3–2.9)	0.56
CA125 (U/ml)	8.9 (7.0–10.9)	9.3 (6.4–14.0)	9.4 (7.2–13.1)	0.13
CA199 (U/ml)	7.7 (5.1–9.9)	8.2 (5.3–14.3)	8.4 (5.2–12.2)	0.16

Data are expressed as M (P25 and P75) or number (%)

*ESD* Endoscopic mucosal dissection, *EMR* Endoscopic mucosal resection, *LARG* Laparoscopic assisted radical gastrectomy, *ORG* Open radical gastrectomy

^*^Fisher’s exact test was used. Angiosis: atherosclerotic lesions of the heart or brain. Family history: the patient had familial gastric cancer in his immediate family

### Comparative analysis of oncologic features

The median tumor sizes in the three groups were 1.5 cm (ESD/EMR), 2.0 cm (LARG), and 2.0 cm (ORG); the tumor diameter of the ESD/EMR group was smaller than those of the other two groups (*P* < 0.05). Cancer of the upper stomach was more common in the ESD/EMR group, while cancer of the lower stomach was more common in the LARG group and ORG group (*P* < 0.05). In terms of pathological types, highly differentiated adenocarcinoma was more common in the ESD/EMR group, while moderately differentiated and poorly differentiated adenocarcinoma were more common in the LARG group and ORG group (*P* < 0.05). In terms of tumor tissue invasion, 108 patients had intramucosal carcinoma in the ESD/EMR group, which was significantly higher than that in the other groups (*P* < 0.05). Surgery had an advantage over ESD/EMR in detecting lymph nodes (*P* < 0.05); the rate of vascular invasion had no difference among all three treatment groups (Table 2).

**Table 2:** Comparative analysis of oncologic features

Characteristic	ESD/EMR (*n* = 139)	LARG (*n* = 108)	ORG (*n* = 170)	*P*
Tumor size (cm)	1.5 (1.0–2.0)	2.0 (1.5–3.0)	2.0 (1.5–3.0)	< 0.01
Location (%)				< 0.01
Upper	77 (55.4)	24 (22.2)	48 (28.2)	
Middle	10 (7.2)	25 (23.2)	28 (16.5)	
Lower	52 (37.4)	59 (54.6)	94 (55.3)	
Infiltration (%)				< 0.01
Mucous	109 (78.4)	52 (48.1)	83 (48.8)	
Submucous	30 (21.6)	56 (51.9)	87 (51.2)	
Pathology (%)				< 0.01
High	83 (59.7)	15 (13.8)	33 (19.4)	
Middle	43 (30.9)	48 (44.4)	59 (34.7)	
Lower	13 (9.4)	45 (41.8)	78 (45.9)	
Lymph node	0	17 (14–21)	16 (12–20)^a^	< 0.01
Lymph node metastasis (%)				< 0.01
Yes	0 (0)	9 (8.3)	23 (13.5)^a^	
No	139 (100)	99 (91.7)	147 (86.5)	
Vascular invasion (%)				0.17*
Yes	1 (0.7)	5 (4.6)	6 (3.5)	
No	138 (99.3)	103 (95.4)	164 (96.5)	

Data are expressed as M (P25 and P75) or number (%)

*ESD* Endoscopic mucosal dissection, *EMR* Endoscopic mucosal resection, *LARG* Laparoscopic assisted radical gastrectomy, *ORG* Open radical gastrectomy

*Fisher’s exact test was used

^a^compared with LARG, *P* > 0.05

### Health economic costs

**Fig. 2:**
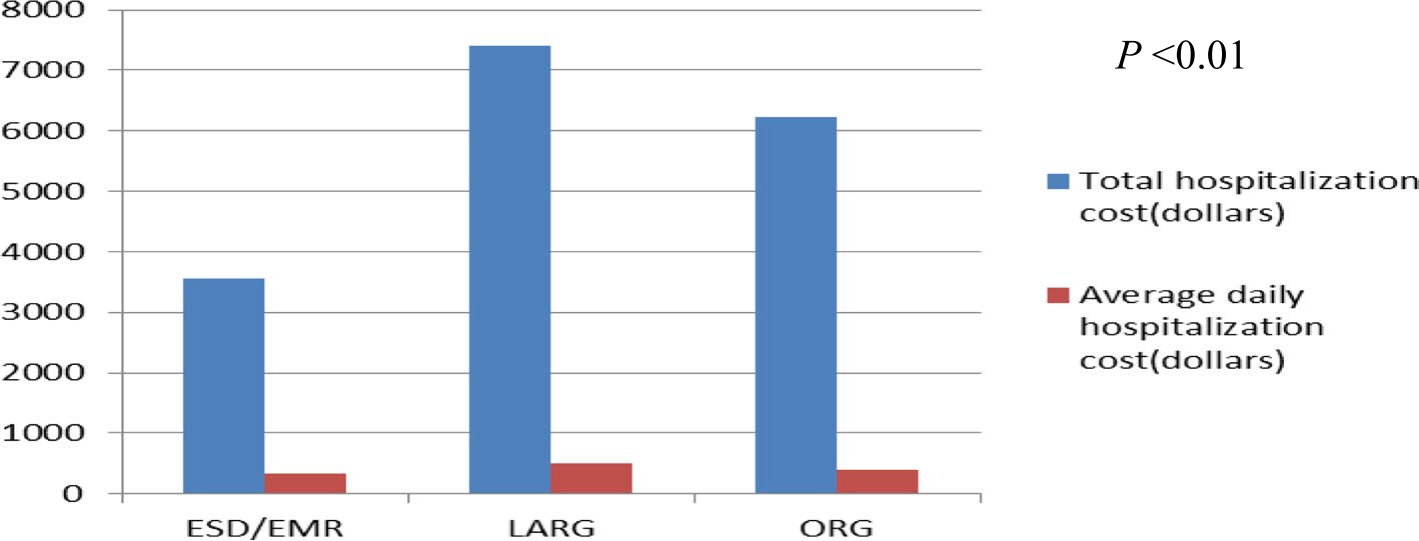
Expenses for hospitalization of gastric cancer patients in the three groups

The total hospitalization cost (dollars) of gastric cancer patients in the three groups were as follows: ESD/EMR, 3,558 (3,219–3,943); LARG, 7,410 (6,570–8,531); and ORG, 6,234 (5,581–7,273). The hospitalization cost of patients in the ESD/EMR group was significantly lower than that in the LARG group and ORG group; the hospitalization cost of patients in the LARG group was the highest, and the difference was significant (*P* < 0.05). The average daily hospitalization cost of the ESD/EMR group was significantly lower than that of the LARG group and ORG group (*P* < 0.05) (Fig. 2 and Table 3).

**Table 3:** Health economic costs

Characteristic	ESD/EMR (*n* = 139)	LARG (*n* = 108)	ORG (*n* = 170)	*P*
Total cost (dollars)	3,558 (3,219–3,943)	7,410 (6,570–8,531)	6,234^b^ (55,81–7,273)	< 0.01
Average cost (dollars)	336 (289–380)	505 (437–568)	409^b^ (331–486)	< 0.01

Data are presented as M (P25 and P75). ESD: endoscopic mucosal dissection

*EMR* Endoscopic mucosal resection, *LARG* Laparoscopic assisted radical gastrectomy, *ORG* Open radical gastrectomy

^b^compared with LARG, *P* < 0.05

### Postoperative short-term clinical effect

The total length of hospital stay, postoperative length of hospital stay, postoperative fluid intake time, and proportion of antibiotic use in the ESD/EMR group were lower than those in the surgical treatment group (*P* < 0.05). However, five patients underwent surgery after ESD/EMR as all of them had residual tissue margin cancer (*P* < 0.05). The total length of hospital stay, postoperative length of hospital stay, postoperative fluid intake time, and proportion of antibiotic use in the LARG group were consistent with those of the ORG group (*P* < 0.05); none of the patients in in either groups had residual tissue margin cancer or underwent reoperation. In terms of the incidence of postoperative incision infection and pulmonary infection, ESD/EMR had lower incidence of complications compared with surgical operation, but only incision infection had statistical difference (*P* < 0.05). No difference was observed in incision infection rate between LARG and ORG. In terms of operation time, ESD/EMR lasted for 90 min, LARG lasted for 250 min, and ORG lasted for 150 min, with significant differences (*P* < 0.05) (Table 4).

**Table 4:** Postoperative short-term clinical effect

Characteristic	ESD/EMR (*n* = 139)	LARG (*n* = 108)	ORG (*n* = 170)	*P*
Hospital stays (d)	11 (9–13)	15 (13–17)	15 (13–18)^a^	< 0.01
Postoperative hospital stay (d)	6 (5–7)	10 (9,11)	10 (8–12)^a^	< 0.01
Fluid intake (d)	3 (3–4)	6 (5–7)	6 (5–7)^a^	< 0.01
Time of operation (min)	90 (70–115)	250 (201–315)	150 (120–180)^b^	< 0.01
Antibiotic (%)	104 (74.8)	105 (97.2)	167 (98.2)^a^	< 0.01
Lesions residue (%)	5 (3.5)	0 (0)	0 (0)	< 0.01*
Referral for surgery (%)	5 (3.5)	0 (0)	0 (0)	< 0.01*
Infection of incision (%)	0 (0)	3 (0)	6 (3.5)^a^	0.04*
Lung infection (%)	0 (0)	2 (0)	4 (2.3)^a^	0.18*

Data are expressed as M (P25 and P75) or number (%)

*ESD* Endoscopic mucosal dissection, *EMR* Endoscopic mucosal resection, *LARG* Laparoscopic assisted radical gastrectomy, *ORG* Open radical gastrectomy

^*^Fisher’s exact test was used

^a^compared with LARG, *P* > 0.05

^b^compared with LARG, *P* < 0.05

### Postoperative complications and recurrence

ESD/EMR was compared with surgery in terms of the incidence of postoperative complications; no significant difference was observed in the distribution of postoperative gastrointestinal bleeding, perforation, and incision hernia between the two treatment groups (*P* > 0.05). Meanwhile, the distribution of significant weight loss (a weight loss of 10% or more compared with that preoperatively) was considered significant (*P* < 0.05). Moreover, the incidence of postoperative emaciation was significantly lesser in the ESD/EMR group compared with that in the surgical group, but no Difference was observed between the LARG and ORG groups. Reoperation was performed in five patients from the ESD/EMR group because of perforation or bleeding, two patients from the LARG group because of bleeding, and three patients from the ORG group because of bleeding, but no difference (*P* > 0.05). During follow-up, 28 patients experienced recurrence, including 12 from the ESD/EMR group, 8 from the LARG group, and 10 from the ORG group, but no difference (*P* > 0.05) (Table 5).

**Table 5:** Postoperative complications and recurrence

Characteristic	ESD/EMR (*n* = 139)	LARG (*n* = 108)	ORG (*n* = 170)	*P*
Bleeding (%)	6 (4.3)	10 (9.3)	17 (0.1)	0.15
Perforation (%)	3 (2.1)	0 (0)	1 (0.6)	0.23*
Emaciation (%)	0 (0)	2 (1.8)	4 (2.3)	0.17*
Weight loss (%)	0 (0)	3 (2.7)	7 (4.1)^a^	0.03*
Incision hernia (%)	0 (0)	0 (0)	2 (1.1)	0.34*
Reoperation (%)	5 (3.5)	2 (1.8)	3 (1.7)	0.61*
Recurrence (%)	12 (8.6)	8 (7.4)	10 (5.9)	0.64

Data are expressed as number (%). ESD: endoscopic submucosal dissection

*EMR* Endoscopic mucosal resection, *LARG* Laparoscopic assisted radical gastrectomy, *ORG* Open radical gastrectomy

^*^Fisher’s exact test was used

^a^compared with the LARG, *P* > 0.05

### Survival analysis comparison

**Fig. 3:**
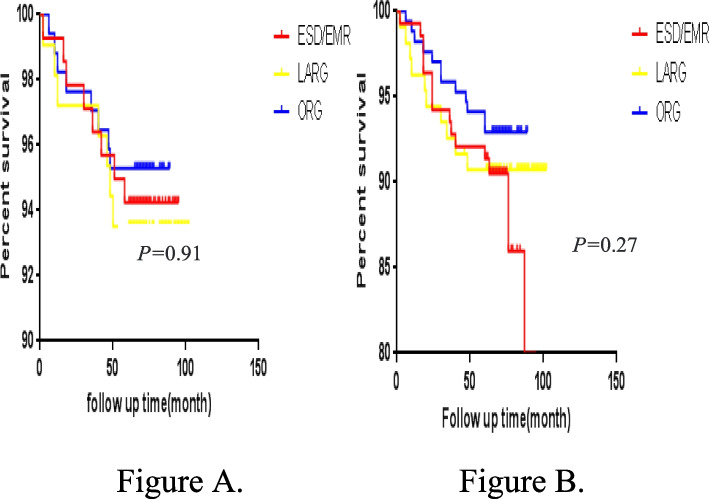
**A** 5-year overall survival curves of gastric cancer patients in the three groups. **B** 5-year disease-free survival curves of gastric cancer patients in the three groups

At the end of follow-up, 8 patients in the ESD/EMR group, 7 patients in the LARG group, and 9 patients in the ORG group died. The 5-year survival rates were 94.2% in the ESD/EMR group, 93.5% in the LARG group, and 94.7% in the ORG group, with no significant difference (*P* > 0.05). The 5-year disease-free survival rates were 89.2% in the ESD/EMR group, 92.6% in the LARG group, and 94.2% in the ORG group, with no significant difference (*P* > 0.05) (Fig. 3).

**Fig. 4:**
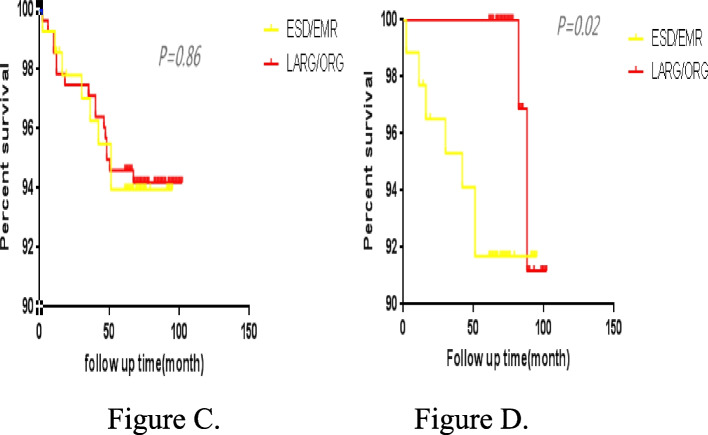
**C** 5-year overall survival curves before the matching propensity score, in the two groups. **D** 5-year overall survival curves after the matching propensity score, in the groups

**Fig. 5:**
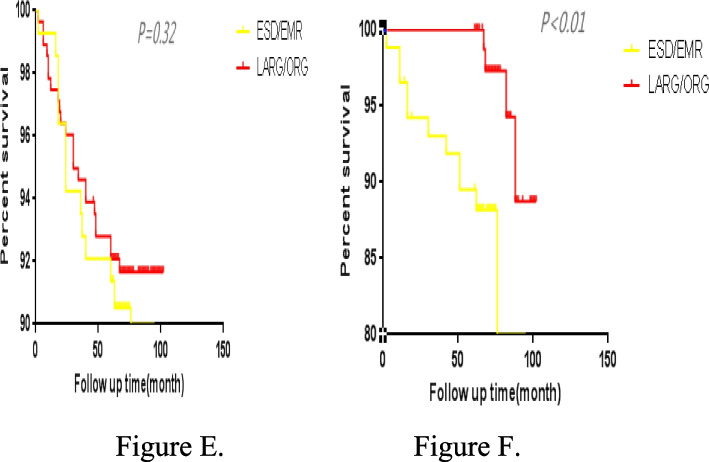
**E** 5-year disease-free survival curves before the matching propensity score, in the two groups. **F** 5-year disease-free survival curves after the matching propensity score, in the two groups

We performed propensity score matching, and finally matched 88 patients in ESD/ EMR group and 88 patients in LARG/ORG group after 1:1 matching. Covariates were sex, age, smoking, drink alcohol, cardiovascular and cerebrovascular diseases, diabetes, family history of cancer, tumor location, tumor size, depth of tumor invasion, pathological differentiation, and vascular invasion. After 1:1 matching, there were no statistical differences in various clinical data between the two groups, as shown in Table 6. After the matching propensity, the results score showed that the LARG/ORG patients had better in 5-year survival rates than the ESD/EMR patients, as shown in Fig. 4C, D. After the matching propensity, the results score showed that the LARG/ORG patients had better in 5-year disease-free survival rates than the ESD/EMR patients, as shown in Fig. 5E, F.

**Table 6:** Comparison of propensity score matching

Characteristic	Before matching			After matching		
	ESD/EMR(139)	LARG/ORG(278)	*P*	ESD/EMR(88)	LARG/ORG (88)	*P*
Sex (%)						0.86
Male	113(81.3)	197(70.8)	0.02	68(77.3)	66(75.0)	
Female	26(18.7)	81(29.2)		20(22.7)	22(25.0)	
Year (%)			0.12			0.86
≥ 60	91(65.4)	160(57.5)		65(73.9)	63(71.6)	
< 60	48(34.6)	118(42.5)		23(26.1)	25(28.4)	
Smoking (%)			0.65			0.83
Yes	24(17.2)	53(19.0)		15(17.0)	13(14.8)	
No	125(82.8)	225(81.0)		73(83.0)	75(85.2)	
Drink (%)			0.73			0.99
Yes	14(10.0)	31(11.1)		6(6.8)	6(6.8)	
No	125(90.0)	247(89.9)		82(93.2)	82(93.2)	
Atherosis (%)			0.85			0.70
Yes	26(18.7)	50(17.9)		16(18.2)	19(21.6)	
No	113(81.3)	228(82.1)		72(81.8)	69(78.4)	
Diabetes (%)			0.99			0.99
Yes	5(3.5)	10(3.5)		2(2.3)	2(2.3)	
No	134(96.5)	268(96.5)		86(97.7)	86(97.7)	
Family history (%)			0.99			0.99
Yes	4(2.9)	8(2.9)		3(3.4)	3(3.4)	
No	135(97.1)	270(97.1)		85(96.6)	85(96.6)	
Vascular invasion (%)			0.06			0.99
Yes	1(0.7)	11(3.9)		1(1.1)	1(1.1)	
No	138(99.3)	267(96.1)		87(98.9)	87(98.9)	
Location (%)			< 0.01			0.21
Upper	77(55.3)	72(25.9)		45(51.1)	40(45.5)	
Middle	10(7.2)	53(19.0)		8(9.1)	16(18.2)	
Lower	52(37.5)	153(55.1)		35(39.8)	32(36.4)	
Tumor size (%)			< 0.01			0.65
≥ 2 cm	41(29.4)	188(67.6)		39(44.3)	43(48.9)	
< 2 cm	98(71.6)	90(32.4)		49(55.7)	45(51.1)	
Differentiation (%)			< 0.01			0.89
High	83(59.7)	48(17.2)		40(45.5)	42(47.7)	
Middle	43(30.9)	107(38.5)		35(39.8)	35(39.8)	
Lower	13(9.4)	123(44.3)		13(14.8)	11(12.5)	
Infiltration(%)			< 0.01			0.39
Mucous	109(78.4)	135(48.5)		67(76.1)	61(69.3)	
Submucous	30(21.5)	143(51.5)		21(23.9)	27(30.7)

### Univariate and multivariate analyses

A single factor analysis of death in patients with EGC showed that the tumor location (*P* = 0.03), tumor size (*P* < 0.01), depth of invasion (*P* < 0.01), degree of differentiation (*P* < 0.01), and vascular tumor thrombus (*P* < 0.01) were significantly correlated with death (Table 7). Furthermore, multivariate analysis using binary logistic regression model showed that the tumor size (*P* = 0.03), invasion depth (*P* = 0.01), vascular invasion (*P* < 0.01), and degree of differentiation (*P* = 0.01) were risk factors for death in patients with EGC (Table 8). Univariate analysis was performed in each group, and the influencing factors of death were tumor size (*P* < 0.01), infiltration depth (*P* < 0.01), and degree of differentiation (*P* = 0.04) in the ESD/EMR group; sex (*P* = 0.02), infiltration depth (*P* = 0.01), and vascular invasion (*P* = 0.03) in the LARG group; and infiltration depth (*P* = 0.03), degree of differentiation (*P* = 0.03), and vascular invasion (*P* = 0.01) in the ORG group (Table 9). Multivariate analysis using binary logistic regression model showed that the tumor size (*P* = 0.01) and degree of differentiation (*P* = 0.04) were risk factors for death in the ESD/EMR group, the infiltration depth (*P* = 0.01), degree of differentiation (*P* = 0.01) and vascular invasion (*P* = 0.01) were risk factors in the LARG/ORG group (Table 10).

**Table 7:** Single factor analysis of death in patients with early gastric cancer

Characteristic	Survival (*n* = 393)	Death (*n* = 24)	*P*
Sex (%)			0.38
Male	294 (74.8)	16 (66.6)	
Female	99 (25.2)	8 (33.4)	
Year (%)			0.50
≥ 60	235 (59.8)	16 (66.7)	
< 60	158 (41.2)	8 (33.3)	
Location (%)			0.02*
Upper	144 (36.6)	5 (20.8)	
Middle	55 (14)	8 (33.3)	
Lower	194 (49.4)	11 (45.8)	
Tumor size (%)			< 0.01
≥ 2 cm	208 (53)	21 (83.3)	
< 2 cm	185 (47)	3 (16.7)	
Infiltration (%)			< 0.01
Mucous	241 (61.3)	3 (12.5)	
Submucous	152 (38.7)	21 (87.5)	
Differentiated degree (%)			< 0.01
High	128 (32.6)	3 (12.5)	
Middle	145 (36.9)	5 (20.8)	
Lower	120 (30.5)	16 (66.7)	
Vascular invasion (%)			< 0.01
Yes	7 (2)	5 (20.8)	
No	386 (98)	19 (79.2)	
Smoking (%)			0.78*
Yes	72 (18.3)	5 (20.8)	
No	321 (81.7)	19 (79.2)	
Drink (%)			0.99*
Yes	42 (10.7)	3 (12.5)	
No	351 (89.6)	21 (87.5)	
Diabetes (%)			0.99*
Yes	14 (3.6)	1 (4.2)	
No	379 (96.4)	23 (95.8)	
Atherosis (%)			0.27*
Yes	74 (18.8)	2 (8.3)	
No	319 (81.2)	22 (91.7)	
Family history (%)			0.99*
Yes	11 (2.5)	1 (4.1)	
No	382 (97.5)	23 (95.9)	
Lymph node metastasis (%)			0.41*
Yes	29 (7.3)	3 (12.5)	
No	364 (92.7)	21 (87.5)	

Data are presented as number (%)

*Fisher’s exact test was used

**Table 8:** Multivariate analysis of death in patients with early gastric cancer

Characteristic	B	SE	Wals	OR	HR (95% CI)	*P*
Location	0.142	0.32	0.19	1.15	0.61–2.16	0.65
Tumor size	1.48	0.68	4.75	4.40	1.16–16.70	0.03
Infiltration depth	2.19	0.67	10.04	8.45	2.25–31.64	0.01
Differentiated degree	0.95	0.38	6.29	2.59	1.23–5.47	0.01
Vascular invasion	2.31	0.74	9.60	10.09	2.33–43.54	< 0.01
ESD/EMR	1.29	0.88	2.11	3.64	0.63–20.75	0.14

**Table 9:** Single factor analysis of death in the three groups

Characteristic	ESD (*n* = 139)		*P*	LARG (*n* = 108)		*P*	ORG (*n* = 170)		*P*
	Survival (*n* = 131)	Death (*n* = 8)		Survival (*n* = 101)	Death (*n* = 7)		Survival (*n* = 161)	Death (*n* = 9)	
Sex (%)			0.99*			0.02*			0.99*
Male	106 (80.9)	7 (87.5)		74 (73.3)	2 (28.6)		114 (70.8)	7 (77.8)	
Female	25 (19.1)	1 (12.5)		27 (26.7)	5 (71.4)		47 (29.2)	2 (22.2)	
Year (%)			0.99*			0.45*			0.74*
≥ 60	86 (65.6)	3 (37.5)		54 (71.4)	5 (71.4)		95 (59)	6 (66.7)	
< 60	45 (34.4)	5 (62.5)		47 (46.5)	2 (28.6)		66 (41)	3 (33.3)	
Location (%)			0.12*			0.29*			0.05*
Upper	74 (56.5)	3 (37.5)		22 (21.8)	2 (28.6)		48 (29.8)	0 (0)	
Middle	8 (6.1)	2 (25)		22 (21.8)	3 (42.9)		25 (15.5)	3 (33.3)	
Lower	49 (37.4)	3 (37.5)		57 (56.4)	2 (28.6)		88 (54.7)	6 (66.7)	
Tumor size (%)			< 0.01*			0.41*			0.27*
≥ 2 cm	34 (26)	7 (87.5)		65 (64.4)	6 (85.7)		109 (67.7)	8 (88.9)	
< 2 cm	97 (74)	1 (12.5)		36 (35.6)	1 (14.3)		52 (32.3)	1 (11.1)	
Infiltration depth (%)			< 0.01*			0.01*			0.03*
Mucous	107 (81.7)	2 (25)		65 (64.4)	0 (0)		82 (50.9)	1 (11.1)	
Submucous	24 (18.3)	6 (75)		36 (35.6)	7 (100)		79 (49.1)	8 (88.9)	
Differentiated degree (%)			0.04*			0.29*			0.03*
High	80 (61.1)	3 (37.5)		15 (14.9)	0 (0)		33 (20.5)	0 (0)	
Middle	41 (31.3)	2 (25)		46 (45.5)	2 (28.6)		58 (36.0)	1 (11.1)	
Lower	109 (7.6)	3 (37.5)		40 (39.6)	5 (71.4)		70 (43.5)	8 (88.9)	
Vascular invasion (%)			0.05*			0.03*			0.03*
Yes	0 (0)	1 (12.5)		3 (3.0)	2 (28.6)		4 (2.5)	2 (22.2)	
No	131 (100)	7 (87.5)		98 (97.0)	5 (71.4)		157 (97.5)	7 (77.8)	
Smoking (%)			0.62*			0.99*			0.67*
Yes	22 (16.8)	2 (25)		21 (20.8)	1 (14.3)		29 (18)	2 (22.2)	
No	109 (83.2)	6 (75)		80 (79.2)	6 (85.1)		132 (82)	7 (77.8)	
Drink (%)			0.99*			0.99*			0.24*
Yes	14 (10.7)	0 (0)		12 (11.9)	1 (14.3)		16 (9.9)	2 (22.2)	
No	117 (89.3)	8 (100)		89 (88.1)	6 (85.7)		145 (90.1)	7 (77.8)	
Diabetes (%)			0.99*			0.18*			0.99*
Yes	5 (3.8)	0 (0)		2 (2.0)	1 (14.3)		7 (4.3)	0 (0)	
No	126 (96.2)	8 (100)		99 (98.0)	6 (85.7)		154 (95.7)	9 (100)	
Atherosis (%)			0.35*			0.99*			0.99*
Yes	26 (19.8)	0 (0)		18 (17.8)	1 (14.3)		30 (18.6)	1 (11.1)	
No	105 (80.2)	100 (100)		83 (82.2)	6 (85.7)		131 (81.4)	8 (88.9)	
Family history (%)			0.21*			0.99*			0.99*
Yes	3 (2.3)	1 (12.5)		3 (3.0)	0 (0)		5 (3.1)	0 (0)	
No	105 (80.2)	7 (87.5)		98 (97.0)	100 (100)		155 (98.9)	9 (100)	
Lymph metastasis (%)									0.35*
Yes	0	0		8 (7.9)	1 (14.3)	0.46*	21 (13.0)	2 (22.2)	
No	131	8		93 (92.1)	6 (83.7)		140 (87.0)	7 (77.8)	

Data are expressed as number (%). ESD: endoscopic submucosal dissection

*EMR* Endoscopic mucosal resection, *LARG* Laparoscopic assisted radical gastrectomy, *ORG* Open radical gastrectomy

^*^Fisher’s exact test was used

**Table 10:** Multivariate analysis of death in the two groups

Characteristic	ESD/EMR(*n* = 139)		LARG/ORG(*n* = 278)	
	HR	*P*	HR	*P*
Sex	0.36–59.71	0.23	0.21–2.25	0.54
Location	0.97–11.58	0.05	0.27–1.37	0.23
Tumor size	1.96–316	0.01	0.81–22.41	0.08
Infiltration depth	1.03–50.22	0.04	1.54–98.15	0.01
Differentiated degree	0.62–6.31	0.24	1.52–25.25	0.01
Vascular invasion	0	0.99	1.67–45.35	0.01

*ESD* Endoscopic submucosal dissection, *EMR* Endoscopic mucosal resection, *LARG* Laparoscopic assisted radical gastrectomy, *ORG* Open radical gastrectomy

## Discussion

With the continuous development of imaging technology, the diagnosis rates of EGC have increased up to 10%–15% in China and 70% in Japan [6]. The prognosis of EGC is significantly better than that of advanced-stage gastric cancer, and the 5-year survival rate is more than 90% [7]. The treatment of EGC can be divided into endoscopic treatment and surgical treatment. Endoscopic treatment includes ESD and EMR, with the latter comprising LARG and ORG. Some complications may occur after gastric cancer surgery, such as gastric stump cancer, reflux gastritis, wasting, etc., which can seriously affect the patients’ quality of life [8–10].Therefore, EGC is treated with radical surgery with preservation of the gastrointestinal physiological function [11]. According to the reports of relevant studies, ESD was performed for EGC; the overall resection rate was 94.9%, and the complete resection rate was 94.7% [12]. The current domestic EGC treatment remains controversial. This study used clinical data from retrospective cohort studies; in this study, 417 patients with EGC were divided into ESD/EMR group, LARG group, and ORG group. Relevant clinical data were analyzed in order to provide reference for the rational selection of treatment modalities for EGC. We screened patients with early gastric cancer according to the absolute indications and expanded indications of ESD/EMR treatment. For patients with early gastric cancer within these indications, although ESD/EMR is the standard treatment, radical surgery is also the main surgical treatment at present, especially LARG, which is also one of the surgical methods recommended by the Japanese guidelines. Despite the increasingly widespread application of ESD/EMR surgery, the concept that traditional surgery can thoroughly clean lymph nodes remains deeply rooted. Many people believe that traditional surgery can bring them better prognosis, so they actively choose traditional surgery, including open and laparoscopic radical gastrectomy for gastric cancer.

No statistical differences were found in the gender, age, body weight, preoperative blood routine indexes, preoperative blood tumor markers, smoking history, and drinking history among the three treatment groups, thus suggesting that the baseline data were comparable. In the ESD/EMR group, the upper gastric carcinoma was more common, most of the patients had highly differentiated adenocarcinoma, and the tumor diameter was significantly smaller than that of the surgical operation groups. In terms of health economic costs, the related hospitalization costs in the ESD/EMR group were significantly lower than those in the surgical operation groups, while the hospitalization costs of the LARG group were the highest cost; this finding is consistent with those of studies in other countries [13, 14]. The total hospitalization days and postoperative hospitalization days of the ESD/EMR group were lesser than those of the surgical operation group, and previous studies have found similar results [14, 15].The postoperative fluid intake time and proportion of antibiotic use in the ESD/EMR group were significantly lower than those in the surgical operation groups, this may be related to the higher rate of trauma after gastrectomy and antibiotic prophylactic treatment during the perioperative period. Related studies reported that a bleeding rate of 7% and a perforation rate of 4% after ESD/EMR [16–18]. In our study, only 6 patients experienced bleeding and 3 experienced perforation after undergoing ESD/EMR, which were lower than those reported in previous studies; these findings suggest that the ESD/EMR technology has already matured after decades of rapid development. In the study, only 6 patients experienced bleeding in the ESD/EMR group, 27 in the surgical operation group, there were more patients in the surgical group. In our study, five patients required further surgical treatment after undergoing ESD/EMR because of residual margin. However, the incidence of postoperative abdominal distention and infection of incision was higher in the ORG group (*P* < 0.05). Reoperation was performed in five patients from the ESD/EMR group because of perforation or bleeding, two patients from the LARG group because of bleeding, and three patients from the ORG group because of bleeding, but the difference in distribution was not significant (*P* > 0.05).

Previous literature had reported that the recurrence rate of tumors after ESD/EMR is 8.2%–14%, while the recurrence rate of gastric cancer after radical gastrectomy is 0.6%–3.3% [19, 20]. This study found no significant difference in the tumor recurrence rates among all treatment groups. During follow-up, recurrence cases were reported in the three groups, among the 12 patients in the ESD/EMR group, 18 patients in the surgical operation group, no significant difference. Among the 12 patients in the ESD/EMR group, all of them were detected by endoscopy, including 4 cases within 12 months and 8 cases after 12 months. These lesions were all new lesions, most of which appeared near the primary gastric lesions, and the histopathological types were the same as those of the original lesions. Among them, 5 patients had mild abdominal discomfort, such as abdominal pain, abdominal distension, etc., and most of the patients had no obvious symptoms, the invasion depth of recurrent tumors was basically located in the mucosal layer, and most lesions were about 1 cm in diameter. Due to regular follow-up, tumor recurrence was detected in time, 9 patients received ESD treatment again, and 3 patients received lymphadenectomy. However, no lymph node metastasis was found. This study found that the 5-year survival rates of the ESD/EMR, LARG, and ORG groups were 94.2%, 93.5%, and 94.7%, the 5-year disease-free survival rates were 89.2% in the ESD/EMR group, 92.6% in the LARG group, and 94.2% in the ORG, with no significant difference (*P* > 0.05). Some patients showed the 5-year OS rates of 97.5% and 97.0%, respectively, after endoscopic resection and surgery [21]. After survival analysis, we found that there was no significant difference in 5-year OS and DFS after surgery between the endoscopic group and the surgical group for EGC patients. However, our study was a retrospective cohort study, and there were differences in tumor characteristics between groups, such as tumor size, location, differentiation, and depth of invasion, which might affect the results of survival analysis. Considering that the oncologic characteristics of the included EGC patients were unbalanced among the groups, we took the general conditions and oncologic characteristics of the patients as covariables to perform propensity score matching. After matching propensity scores, we found that the 5-year OS of the LARG/ORG group was 96.5%, and that of the ESD/EMR group was 92.0%, showing a significant statistical difference. In addition, the 5-year DFS of LARG/ORG group was significantly better than that of ESD/EMR group. After matching propensity scores, we eliminated confounding factors between the groups, effectively controlled confounding bias, and made baseline data similar between ESD/EMR group and LARG/ORG group, with balanced comparability between the two groups. It should not be ignored that after propensity score matching, we lost some sample size, but the sample size after matching was still representative, and the biggest advantage was that we exclude the influence of confounding bias on survival outcome, so the survival analysis results after matching were reliable. Through propensity score matching, we found that LARG/ORG group had significantly better OS and DFS than ESD/EMR group. Compared with endoscopic treatment, surgical treatment had obvious advantages of wide resection range and thorough dissection of lymph nodes, surgical treatment of EGC can achieve a radical effect, which had a very important impact on the survival of EGC patients. In addition, our study included more patients with extended indications in line with endoscopic therapy guidelines, while the efficacy of ESD/EMR was still controversial. We should be cautious when performing ESD/EMR treatment for those who meet the expanded indications of ESD/EMR treatment for EGC patients.

The univariate analysis showed that the tumor location, tumor size, invasion depth, vascular invasion, degree of differentiation were the risk factors for death; meanwhile, the binary logistics multivariate analysis showed that the tumor size, invasion depth, vascular invasion, and degree of differentiation were the risk factors, but the tumor location and ESD/EMR treatment were not risk factors. A repeat univariate analysis was performed for each group, and the influencing factors of death were the tumor size, infiltration depth and degree of differentiation in the ESD/EMR group; the sex, infiltration depth, and vascular invasion in the LARG group; and infiltration depth, degree of differentiation, and vascular invasion in the ORG group. Multivariate analysis using binary logistic regression model showed that the tumor size and infiltration depth were risk factors for death in the ESD/EMR group, the infiltration depth, degree of differentiation and vascular invasion were risk factors in the LARG/ORG group.

In the development of endoscopic treatment of gastric cancer in Japan, EMR has not reached the therapeutic value that is sufficient to replace surgical treatment, but the emergence of ESD has changed this situation [16, 22].Currently, the indications for ESD/EMR have included undifferentiated carcinoma (no lymphovascular invasion and no ulceration) of less than 2 cm in the mucosa and differentiated adenocarcinoma (no lymphovascular invasion and no ulceration) of less than 3 cm in the submucosa. However, there were still different opinions on the effectiveness of the expanded criteria. According to Kang et al., the rate of mucosal lymph node metastasis reached 1.4%, while the rate of submucosal lymph node metastasis was 15%, even if the ESD criteria are fully met [23]. Holscher et al. reported that tumors with a deep mucosal infiltration (≥ 2 cm) had significant risk of lymph node metastasis [24], regardless of histology. Other related studies have also reported that the lymph node metastasis rate of intramucosal carcinoma was 2%–3%, while the lymph node metastasis rate of submucosal carcinoma was 15%–20% [25–27]; among the 278 patients who underwent radical surgery, lymph node metastasis was found in 11% of the patients after surgery. Therefore, a more accurate and consistent method for predicting lymph node metastasis is needed to support the necessity of performing radical resection using EMR/ESD. Although ESD/EMR is associated with less trauma, faster recovery, higher quality of life, and less cost, the risk of lymph node metastasis is higher when using the extension criteria. Our study also found that 5 patients were converted to surgery after ESD/EMR because of residual cancer, while 5 patients were reselected for surgical treatment due to recurrence during long-term follow-up. Lymph node metastasis of intramucosal carcinoma was significantly less common than that of submucosal carcinoma. Hence, patients who meet the absolute indication of ESD/EMR in intramucosal carcinoma should be given priority.

This study has some limitations. 1. It was a retrospective study, compared with a prospective study, with a lower level of evidence; and fewer cases, especially the ESD/EMR group and LARG group. 2. No questionnaire survey on postoperative quality of life was conducted during follow-up due to the abovementioned limitations. In spite of these shortcomings, this study is still meaningful. A comprehensive and systematic comparative study was conducted among the three groups of gastric cancer patients, covering general data, oncologic characteristics, perioperative indicators, short-term and long-term postoperative complications, etc.

In conclusion, ESD/EMR has quick postoperative recovery and low economic cost compared with surgery, but LARG/ORG may be superior to ESD/EMR for EGC patients in the 5-year OS and DFS rates. However, if preoperative metastatic lymph nodes cannot be completely excluded, radical surgery can avoid potential risks.

## Data Availability

Data will be made available on request.
